# Biocontrol of Pathogen Microorganisms in Ripened Foods of Animal Origin

**DOI:** 10.3390/microorganisms11061578

**Published:** 2023-06-14

**Authors:** Josué Delgado, Micaela Álvarez, Eva Cebrián, Irene Martín, Elia Roncero, Mar Rodríguez

**Affiliations:** Higiene y Seguridad Alimentaria, Instituto de Investigación de Carne y Productos Cárnicos (IProCar), Facultad de Veterinaria, Universidad de Extremadura, Avda. de las Ciencias s/n, 10003 Cáceres, Spain; jdperon@unex.es (J.D.); maalvarezr@unex.es (M.Á.); evcebrianc@unex.es (E.C.); iremartint@unex.es (I.M.); eroncerob@unex.es (E.R.)

**Keywords:** dry-cured meat, ripened cheese, pathogens, molds, yeasts, lactic acid bacteria, essential oils

## Abstract

Ripened foods of animal origin comprise meat products and dairy products, being transformed by the wild microbiota which populates the raw materials, generating highly appreciated products over the world. Together with this beneficial microbiota, both pathogenic and toxigenic microorganisms such as *Listeria monocytogenes*, *Salmonella enterica*, *Staphylococcus aureus*, *Clostridium botulinum*, *Escherichia coli*, *Candida* spp., *Penicillium* spp. and *Aspergillus* spp., can contaminate these products and pose a risk for the consumers. Thus, effective strategies to hamper these hazards are required. Additionally, consumer demand for clean label products is increasing. Therefore, the manufacturing sector is seeking new efficient, natural, low-environmental impact and easy to apply strategies to counteract these microorganisms. This review gathers different approaches to maximize food safety and discusses the possibility of their being applied or the necessity of new evidence, mainly for validation in the manufacturing product and its sensory impact, before being implemented as preventative measures in the Hazard Analysis and Critical Control Point programs.

## 1. Introduction

Ripened foods of animal origin can be divided into two main groups: meat products (dry-cured pieces and dry-cured fermented products, the latter commonly made via mincing and stuffing) and dairy products (mainly, ripened cheeses). These traditional foodstuffs are well-known and highly appreciated all over the world.

The environmental conditions throughout the ripening process of which these animal-derived products undergo favor the growth of diverse microbial populations that deeply contribute to their transformation. Most of these microorganisms, such as some molds, yeasts, gram-positive catalase-positive cocci (GCC+) and lactic acid bacteria (LAB) [1,2,3,4,5,6] have a positive impact on the development of the required sensory characteristics. The presence of this beneficial microbiota is not unique in these products since they are generally exposed to the wild microbiota of the processing environment. Additionally, this processing rarely entails any sanitizing step; pasteurization in artisanal dairy products could be performed, although it is not common; for meat products, however, it is negligible. Thus, the contamination of these products with pathogenic or toxigenic microorganisms usually leads to their development during the industrial ripening of these meat products. In this respect, bacteria such as *Listeria monocytogenes*, *Salmonella* spp., *Staphylococcus aureus*, *Clostridium perfringens*, *Clostridium botulinum*, *Bacillus cereus* and *Escherichia coli* are of most concern for ripened foods of animal origin [7,8,9,10,11,12,13,14,15,16,17,18]. In addition, fungal growth on their surface is also common, especially on dry-cured meat products [19,20,21]. Although these fungi similarly lead to the development of positive sensory characteristics [4,22], some of them can produce mycotoxins such as ochratoxin A (OTA), aflatoxins (AFs), cyclopiazonic acid (CPA), sterigmatocystin (STG) and citrinin (CIT) [23,24,25]. 

Since 2020, and through the Rapid Alert System Feed and Food (RASFF) developed by the European Union, a total of 345 notifications of pathogenic microorganisms in milk and dairy products as well as in meat and meat products were detected, along with 1 notification of the presence of mycotoxins in raw milk. Among all of them, 234 related to meat products, (28 of which were in dry-cured meat products), and 111 related to pathogenic microorganisms detected in cheese [26]. Therefore, it is crucial to understand and apply strategies that allow the survival of beneficial microorganisms in order to transform the products to be capable of hindering pathogenic or spoilage microorganisms. Furthermore, these strategies are considered as preventive measures within the Hazard Analysis and Critical Control Point (HACCP) programs of the industry. 

There are several effective treatments that could be applied in ripened foods of animal origin, including physical methods (heat treatments, ionizing radiation, high hydrostatic pressures) and chemical preservatives (organic acids, antifungal compounds, nitrates and nitrites). However, these methods are not always compatible with the ripening process and can have a negative impact on the organoleptic characteristics of the final product since they are not selective, i.e., they can damage the beneficial microbiota of these ripened foods [27]. In addition, it has been reported that the inappropriate or continuous application of different synthetic compounds to control the pathogenic microorganisms could favor their resistance [28]. Moreover, consumers currently demand clean label products that are free from chemical additives and preservatives [29]. Therefore, preventive measures, such as strategies to control pathogenic or spoilage microorganism in ripened foods of animal origin, are currently based on the use of biocontrol agents (BCAs) of either microbial [30,31,32,33,34,35,36,37,38,39] or plant-based origin, such as essential oils (EOs) and spices [40,41,42,43,44,45]. These treatments should have a low environmental impact and a neutral or positive influence on the sensory properties of the ripened foods of animal origin, since their organoleptic characteristics are well defined and highly appreciated by consumers. 

In the present work, the main pathogenic and toxigenic microorganisms that are able to develop in the different types of ripened foods of animal origin will be studied. Promising BCAs that are being used to reduce or eliminate them, as well as the challenges that their application entails and remain to be solved, are considered. 

## 2. Pathogenic Bacteria in Ripened Foods and Biocontrol Strategies

### 2.1. Pathogenic Bacteria

It is important to monitor the growth of the microorganisms described below as they can grow in ripened food of animal origin that has not undergone any sanitizing process. Thus, these products will be mostly considered as ready-to-eat (RTE). The presence of the following described pathogens in these foods pose a considerable risk which must be controlled using different strategies, with BCAs being among the most appropriate ones.

#### 2.1.1. *Listeria monocytogenes*

*L. monocytogenes* is a gram-positive, motile, facultative anaerobic bacterium that causes listeriosis. This foodborne pathogen can present flu-like symptoms such as fever, fatigue and gastrointestinal symptoms (nausea, vomiting and diarrhea). However, it can cause more severe life-threatening infections in high-risk population groups, such as septicemia, meningitis, meningoencephalitis, spontaneous abortion, still birth or fetal infection [46,47,48]. This pathogen has the capacity to pass three important barriers in the human host, namely the intestinal epithelium, the blood–brain barrier and the placenta, and subsequently disseminate to other organs [49]. 

There have been sixty-seven notifications since 2020 for milk and milk products in the whole of the European Union according to RASFF, of which about 50% were notifications in France for raw milk cheeses. Only eight notifications about the presence of *L*. *monocytogenes* in dry-cured meat products arose from diverse countries from the European Union [26]. In 2021 and 2022, among the total risks identified via the RASFF system, notifications due to the presence of *L. monocytogenes* in soft cheeses comprised 33.5% of the cases [26]. Specifically, in 2022, within biological contaminants, notifications due to *L. monocytogenes* accounted for 31.5%, with cheeses made with raw milk being involved in 57% of cases, with 100% of them originating in Europe and with France being the origin in 45.5% of cases. 

#### 2.1.2. *Salmonella enterica*

*S. enterica* is a gram-negative, motile bacterium that causes salmonellosis. Clinical manifestations are usually fever, weight loss, headache, lethargy, malaise, gastrointestinal bleeding, decreased white blood cells and platelets, and even neurological complications [46].

*S. enterica* invades the intestinal epithelium due to virulence factors that allow for intracellular multiplication in intestinal and immune cells such as macrophages. In this way, the bacteria reach the bloodstream, evading immune activity, and eventually reach the liver, spleen and bone marrow, where they continue to proliferate.

Eight cases of *Salmonella* in ripened cheese and sixteen cases in dry-cured meat products have been identified from 2020 to the present in the European Union [26].

#### 2.1.3. *Staphylococcus aureus*

*S. aureus* is a gram-positive, nonmotile, coagulase-positive and ubiquitous bacterium. It can grow and produce thermostable enterotoxins resistant to digestive enzymes, which are responsible for staphylococcal food poisoning (SFP) [50,51]. The main symptoms of SFP are nausea, vomiting, diarrhea and abdominal pain [52], although more serious and life-threatening infections such as sepsis, necrotizing fasciitis, infective endocarditis, necrotizing pneumonia and toxic shock syndrome [53], although by far less common, are provoked directly by *S. aureus* invasion.

In fact, *S. aureus* has the ability to colonize the skin and mucous membranes of humans and warm-blooded animals. This bacterium has been isolated from a wide range of foods, such as fermented meat and cheese [54,55]. In addition, *S. aureus* has many virulence factors that enable for colonization, increasing its ability to trigger diseases [56].

#### 2.1.4. *Clostridium botulinum*

*C. botulinum* is a gram-positive, obligatory anaerobic and spore-forming rod bacterium [57]. Ingestion of this preformed toxins can cause botulism with symptoms such as paralysis, nausea, vomiting, abdominal cramps, irritability, drooping eyelids, fatigue, difficulty feeding and swallowing [58].

Among the seven types of botulinum toxins (A to G), types A, B, E and F are well known to cause human illness. The severe form of food poisoning is rare, but it has a significant mortality rate [59].

One case of *C. botulinum* in ripened cheese has been reported since 2020 in the European Union [26].

#### 2.1.5. *Escherichia coli*

*E. coli* is a gram-negative bacterium that is part of the intestinal microbiota. Symptoms are bloody diarrhea, abdominal pain, vomiting, hemorrhagic colitis, hemolytic uremic syndrome with acute renal failure, thrombotic thrombocytopenia purpura and even septicemia [60,61,62]. *E. coli* O157:H7 is one of the best-known serotypes to contain pathotypes that can cause food-borne infection in humans [46]. 

Since 2020, thirty-one reports of *E. coli* in milk and dairy products, with 90% coming from ripened cheese, have been made. Only two cases were reported for the presence of *E. coli* in cured and matured-meat products, namely in dry-cured sausages in Italy [26].

### 2.2. Microbial Biocontrol Strategies

In this section, biocontrol strategies for all pathogenic bacteria gathered in Section 2.1 are presented jointly, since most of them are used to control more than one pathogen, as displayed in Table 1, and this is how it is addressed in most of the reported studies [63,64].

LAB are one of the most interesting groups of microorganisms that can be used as BCAs [74], mainly due to their recognized key role in fermented foods. Additionally, several strains belonging to species that are part of the LAB group have acquired QPS (Qualified Presumption of Safety) status [75]. Members of the genera Lactobacilli and members of the genera *Lactococcus* and *Pediococcus* were the most commonly explored, namely *Latilactobacillus curvatus*, *L. sakei*, *Lactiplantibacillus plantarum*, *Limosilactobacillus fermentum*, *Lactococcus lactis* and *Pediococcus acidilactici* [71,76,77,78]. Furthermore, LAB have frequently been used as starters or protective cultures due to their natural ability to dominate the microbial population of many foods. In fact, they are naturally found due to their ability to produce antimicrobial compounds, such as lactic acid and other organic acids, ethanol, diacetyl, carbon dioxide, hydrogen peroxide or bacteriocins [79,80]. The only approved bacteriocin for its use in certain ripened foods in the European Union is nisin, produced by *L. lactis* [81]. Pediocin, produced by *Pediococcus* is another type of bacteriocin which effectively preserves fermented food products such as meat, sausage products and cheeses [82].

Most of these metabolites act both individually and synergistically against pathogens [83]. It is known that LAB have a higher inhibition efficacy against pathogenic gram-positive bacteria [84]. Martín et al. [85] reported *in vitro* antibacterial activity of LAB isolated from traditional Spanish dry-cured fermented sausages and cheeses against *L. monocytogenes* (Table 1). When the biocontrol capacity of these strains was tested in traditional RTE ripened foods models, two of them were selected (*Lacticaseibacillus casei* 116 and *L. sakei* 205) because they provoked reductions higher than 2 log cycles of *L. monocytogenes* in cheeses and dry-cured fermented sausages models, respectively. In addition, when *L. sakei* 205 was inoculated into dry-cured fermented sausages, *L. monocytogenes* was reduced by 1.77 log CFU g^−1^ at the end of the ripening process of these products [65]. Furthermore, Martín et al. [86] demonstrated that the inoculation of this strain did not modify the sensory characteristics of dry-cured fermented sausages. *L. casei* 116 caused a decrease in *L. monocytogenes* of 2.2 log CFU g^−1^ when it was inoculated in short-ripening traditional cheeses without modifying their sensory properties [66,87]. Margalho et al. [67] indicated that greater than 3 log reduction in *L. monocytogenes* in Brazilian artisanal cheeses can be achieved using *L. plantarum* (1QB77) after 21 days of ripening. However, there have been no studies of sensory modifications due to the addition of this bacterium.

Campagnollo et al. [36] evaluated the inhibitory activity of LAB strains (*Levilactobacillus brevis* 2-392, *L. plantarum* 1-399 and 4 strains of *E. faecalis* (1-37, 2-49, 2-388 and 1-400)) against *L. monocytogenes* in microscale “Minas” Frescal and semi-hard cheese models. They demonstrated that the addition of a pool of LAB with antimicrobial properties resulted in bacteriostatic effects and inactivation of *L. monocytogenes* in “Minas” Frescal and “Minas” semi-hard cheeses, respectively. However, there are no available studies that display whether the inoculation of this pool of bacteria modifies the sensory characteristics of these products.

In dry-fermented Greek sausages, Pragalaki et al. [68] observed a significant inhibition of *L. monocytogenes* in the treatments with *L. sakei* 8416 and *L. sakei* 4413 compared to the untreated control. Furthermore, sausages produced with these LAB cultures obtained the highest scores for all sensory attributes in the study conducted by Baka et al. [88].

Kačániová et al. [69] studied the inhibitory capacity of 130 LAB isolated from “Bryndza” cheese. About 84% of the LAB isolates presented an inhibitory effect against *S. enterica* subsp. *enterica* and *S. aureus* subsp. *aureus*, with *L. lactis* subsp. *lactis* and *Lactiplantibacillus paraplantarum* being the most effective LAB strains to inhibit *S. enterica* subsp. *enterica*. Pragalaki et al. [68] observed a 2.2 log reduction in the *E. coli* O157:H7 population when *L. sakei* 4413 was inoculated in dry-fermented Greek sausage.

*E. coli* and *S. aureus* have been frequently studied as pathogens to be inhibited in ripened foods of animal origin. Inhibition of *S. aureus* growth by LAB has been related with the production of different compounds such as organic acids and bacteriocins, changes in redox potential, or combined effects of environmental stressors [89]. In a traditional dry-fermented sausage “suçuk”, the presence of *L. sakei* and *Staphylococcus carnosus* decreased *S. aureus* numbers from the first day on, and no *S. aureus* growth was observed [70]. In the presence of *P. acidilactici*, *L. curvatus* and *Staphylococcus xylosus*, a sub-inoculation level of *S. aureus* counts was determined from day 3 onwards. These results show that both starter culture preparations can reduce the growth of *S. aureus* at the initial ripening temperature [70]. Margalho et al. [67] studied a strain of *L. plantarum* (1QB77) with production of antimicrobial compounds isolated from a Brazilian artisan cheese (Minas Gerais), which showed an inhibition of *S. aureus* of approximately 2.3 log CFU g^−1^ in a microscale Cheeses model (microcheese).

The inoculation of *L. plantarum* PCS20 and *L. delbrueckii* DSM 20074 in fermented salami was able to reduce the levels of *C. perfringens* and *Clostridium* spp. by 2.0 and 1.5 log CFU g^−1^, respectively [71].

Excluding this report, there are scarce studies of BCAs being used against *C. botulinum* in these products; this is probably because of its development requirements—it is relatively easy to control with an HACCP using obstacle theory with a combination of a_w_, temperature and pH in each of the phases of the processing of these products [90]. Additionally, in meat products, the usual inclusion of nitrifying salts (sodium nitrate, potassium nitrate, potassium nitrite and sodium nitrite) confers a high protection against *Clostridium* species [91]. A novel and interesting field deserves to be explored in relation to the substitution of these salts via efficient clean-label strategies, since these preservatives have been related to colorectal cancer [92].

Concerning the use of yeasts as BCAs against pathogenic bacteria, some studies have shown the inhibitory effect of *Debaryomyces hansenii*, *Candida* spp., *Geotrichum candidum* and *Pichia* spp. against certain pathogenic microorganisms and, specifically, against *L. monocytogenes* [93,94]. However, most studies about yeasts with antibacterial activity are not carried out on dry-cured fermented food matrices. *D. hansenii* is one of the main yeasts found in dry-cured fermented meat products and cheeses because it is salt tolerant [3,95]. Nevertheless, Alía et al. [33] showed that *D. hansenii* 258H presented a limited action and even boosted *L. monocytogenes* growth in dry-cured ham slices. They also observed the upregulation of some key virulence genes and an unpleasant appearance of the product. Additionally, most of the research about the antibacterial activity of yeasts in ripened dairy products was executed using commercial culture media [96,97,98]. One study focusing on “Tilsit” cheese showed that the yeast *Pichia norvegensis* achieved a reduction of 1.5 log units (CFU cm^−2^) of *L. monocytogenes* in the product [95]. To summarize, the lack of studies on food matrices shows the need for further works focusing on how the yeasts act during processing and if they alter the technological or sensory characteristics.

### 2.3. Plant-Derivative Biocontrol Agents

Another biocontrol method to prevent the presence of pathogens is the use of plant derivatives, of which the most reported are EOs [99]. These EOs are aromatic and volatile oily extracts obtained from plant materials, including flowers, buds, roots, bark and leaves; they are composed of a mixture of phenylpropenes, terpenes and other volatile components such as thymol, carvacrol and eugenol, which are known for their high antimicrobial capacity [100,101]. The majority of EOs are safe for consumer use when used at the proper concentrations and have been generally recognized as safe (GRAS) [102]. The hydrophilic or lipophilic properties of EO constituents, type of microorganism studied and structure of their cell wall are the factors that affect the Eos’ antimicrobial activity [99]. Their application depends on the sensory impact of EOs that has been reported as one of the most negative aspects of their use [42].

The presence of *Juniperus communis* L. EO (0.01; 0.05 and 0.10 µL g^−1^) in fermented sausages inhibited the growth of foodborne pathogens such as *E. coli*, *L. monocytogenes* and *Salmonella* spp. and sulfite-reducing clostridia, although concentrations of more than 0.10 µL g^−1^ had an untypical flavor [72]. In another dry-cured sausage, ‘Chouriço de vinho’, the antimicrobial effect of EOs from herbs and spices traditionally used in seasoning against *Salmonella* spp., *L. monocytogenes* and *S. aureus* was assessed [42]. The bay, garlic, nutmeg, oregano, rosemary and thyme EOs at 0.005% (sensory-acceptable as assessed by consumers) reduced the counts of *Salmonella* spp. and *L. monocytogenes* in the first steps of drying. However, the thyme EO was the only one that completely inhibited the presence of *S. aureus* after 21 days [42]. Cinnamon, pomegranate and strawberry extracts were able to reduce the growth of *L. monocytogenes* in a study conducted on a cured ham-based medium. Cinnamon at 1% concentration was the most effective, reducing up to 3 log CFU mL^−1^ [73]. The incorporation of oregano EO in alginate films applied to slices of ham resulted in a reduction of up to 2.5 log CFU g^−1^ of *L. monocytogenes* [103]. Tea tree oil at a concentration of 0.25% was able to completely inhibit the growth of *E. coli* through several mechanisms of action [104]. On the other hand, *S. aureus* growth has been shown to be reduced, even at low concentrations of T-cadinol, which is present in a variety of essential plant oils and can lead to disintegration of the cell envelope and leakage of cytoplasm [105]. Ethanolic extract of rosemary leaves showed antimicrobial activity against *Shigella sonnei*, *Salmonella* Typhimurium and *L. monocytogenes* bacteria [106]. Rosemary EO showed inhibition and a bactericidal effect *in vitro* against *S. aureus* with a minimum bactericidal concentration of 5 μL mL^−1^ [107].

BCAs for the control of pathogenic bacteria in dry-cured ham and jerky have been little studied since these products are considered safe products due to their low water activity (a_w_) and salt content [108]. The inactivation of pathogens during cured ham processing has been demonstrated [109].

## 3. Pathogenic Yeasts in Ripened Foods and Biocontrol Strategies

### 3.1. Pathogenic Yeasts

Increasing interest exists in the biodiversity and ecology of yeasts in relation to various food products. This has been driven by the realization that yeasts can interact with other yeast species, as well as with other microorganisms in different ecosystems. These interactions may affect the roles that these fungi play in food [110]. The presence of yeasts in ripened foods, as well as their interaction with their autochthonous microbiota, is in most cases directly related to the improvement in the organoleptic characteristics of the final product [111,112]. In dry-cured meat products, both the proliferation of yeasts during the curing process and the addition of yeasts as starter cultures lead to improvements in texture and the production of pleasant volatile compounds [113]. All this is due to yeast metabolic processes of meat constituents, such as lipids and proteins [114]. As in meat products, yeasts are capable of deploying high metabolic activity in dairy products. Due to the variety of cheeses made from different types of milk with different maturation times, there are great physicochemical differences, which means that the microbiota, and in particular the kind of yeasts, vary from cheese to cheese.

*D. hansenii* is the predominant yeast species observed during the ripening of most cheeses [114], being present in 79% of all the cheeses in the study performed by Banjara et al. [115]. However, strains of *Saccharomyces cerevisiae*, *Yarrowia lipolytica*, *G. candidum* and *Kluyveromyces marxianus* have also been reported in cheese, although to a lower extent [113,114,115]. This is due to the aptitude of dairy yeasts for growing in the presence of a high salt concentration, low pH and low a_w_ as well as its ability to metabolize lactic and citric acids [116,117,118]. On the other hand, the 91.9% of strains isolated from high moisture soft cheeses were classified as *Geotrichum* species. [119] (Table 2).

*D. hansenii* is also the dominant species in sausage manufacturing, being found at every manufacturing stage. *Trichosporon ovoides*, *Y. lipolytica*, *Candida intermedia*/*curvata, C*. *parapsilosis*, *C. zeylanoides* and *Citeromyces matritensis* are also present in Spanish fermented sausages, with most of them being psychrotrophic. *C. intermedia*/*curvata*, *C. matritensis*, C*. zeylanoides* and *T. ovoides* were detected only at the first stages of the sausage manufacturing process [120]. In Parma dry-cured ham, yeast species such as *D. hansenii*, *Torulopsis candida* and *Torulopsis famata* have been proved to be the predominant species. Studies of Spanish dry-cured ham showed that the yeast population profile changes significantly during processing. *C. zeylanoides* was the main species at the fresh stage (more than the 90% of isolates), but *D. hansenii* dominated the yeast population after the post-salting stage [121,122].

While yeasts are rarely associated with foodborne infections, a few studies have shown the presence of medically relevant yeast species in various cheeses. The presence of these fungi in some types of cheeses might be a regular cause of both economic and public health problems. Examples of yeasts with the ability to cause these problems include *Candida* spp., *K. marxianus*, *G. candidum*, *D. hansenii* and *Pichia* spp. [123], although we focused on a narrow population segment. *Candida* spp. are part of the normal human microbiota. They are commensal in healthy individuals but become pathogenic when the host’s defence system is compromised, causing conditions ranging from superficial mucosal to life-threatening systemic infections [117]. The genus *Candida* contains 163 species found in different ecosystems. Diseases are caused by some species such as *C. albicans*, *C. tropicalis*, *C. krusei*, *C. glabrata*, *C. guilliermondii* and *C. parapsilosis* [124]. To the general population, yeasts do not cause serious infections, though some species, such as *C. albicans* and *Cryptococcus neoformans*, are opportunistic pathogens that may cause infections in various organ systems, as well as general fungemia [116]. *C. albicans*, *C. glabrata*, *C. parapsilosis* and *C. tropicalis* are responsible for about 95% of *Candida* blood stream infections, although the vast majority of *Candida* spp. are not pathogenic [125]. It is known that contamination can occur due to a lack of attention to proper hand hygiene during milk production, or even due to improper cleaning of tools used to process milk and its derivatives [110,126].

Pathogenic species belonging to the genus *Candida* rarely occur in cheese. For instance, several *Candida* species, including *C. albicans*, have been found in cheese brine [127], but never in ripened cheese. However, *Issatchenkia orientalis* (teleomorph of *C. krusei*), *Clavispora lusitaniae* (teleomorph of *C. lusitaniae*) and *Candida rugosa* were seldom detected in cheese. There are some rare occurrences of species such as *Candida famata*/*D. hansenii* or *C. krusei*/*I. orientalis* in cheese [118]. When 120 samples from traditional Egyptian dairy products were analyzed, yeasts belonging to the *Candida* genus were identified [110] (Table 2). This raises the possibility that dairy products may be carrying pathogenic yeasts [110]. *C. albicans* was not reported in other analyses of yeast populations in cheese, but another opportunistic pathogenic yeast was reported in “feta” cheese [128] (Table 2). Although it has been found in brine [127], the source of these pathogenic yeasts in dairy products is not well known.

In a study analysing the microbial population of cheese, yeast species isolated from cheese were identified as *C. parapsilosis*, *Candida catenulata*, *Y. lipolytica*, *Rhodotorula glutinis* and *Trichosporon* species. *C. parapsilosis*, *C. catenulata* and *Trichosporon* spp. were also found in raw milk from different species and in several types of cheese. This is a public health concern, as it suggests that these species may survive some kind of cheese-making treatments and spread within the human population [129]. From the analysis of 45 artisanal cheese samples, a total of 251 *Candida* strains were isolated [126] (Table 2).

Candida non-albicans species were responsible for an increase in the proportion of cases of fungemia and other complex cases of candidiasis [130,131,132]. *C. parapsilosis*, isolated from some cheeses, is an emerging human pathogen capable of causing invasive candidiasis, but infection due to consumption of contaminated food has not been documented (Table 2). *Y. lipolytica*, isolated from cheese, is also an emerging opportunistic pathogen, although cases are rare [115] (Table 2). Some large-scale studies confirm that *Y. lipolytica* seldom causes infections. Only 4 isolates of *Y. lipolytica* were present among 6082 isolates from blood stream infections in 250 medical centers from 32 countries between 1992 and 2001 [133]. *C. intermedia*, rarely reported as a human pathogen, has been reported as one of the most predominant yeast species in some cheeses in which NaCl concentrations range from 2% to 8% (*w*/*v*) (Table 2).

**Table 2:** The pathogenic capacity of yeasts isolated from different types of cheeses.

Type of Cheese	Yeast	Pathogenic Capacity	Reference
Cottage and cream cheese	*Geotrichum* species	No	[119]
Domiati and Kariesh cheese	*Candida albicans* *Candida lusitaniae* (teleomorph of *Clavispora lusitaniae*) *Candida catenulata*	Yes	[110]
Feta cheese	*Candida tropicalis*	No	[128]
Smeared cheese	*Issatchenkia orientalis* (teleomorph of *Candida krusei*) *C. lusitaniae* (teleomorph of *C. lusitaniae*) *Candida rugosa* *Candida famata* (teleomorph of *Debaryomyces hansenii*)	Yes	[134]
Several types of cheeses	*Candida parapsilosis*, *C. catenulata*, *Yarrowia lipolytica*, *Rhodotorula glutinis* and *Trichosporon* species	Yes	[129]
Artisanal cheese	*C. albicans* and 97.6% as *Candida* non-albicans, distributed in 79.3% of *C. krusei*, 12.3% of *C. glabrata* and 6.0% of *C. tropicalis*	No	[126]
Swiss-type blue cheese, Goat’s milk Cheddar	*C. parapsilosis*	No	[115]
Manteca (Italian cheese)	*C. parapsilosis*	No	[115]
Washed rind cheese	*Y. lipolytica*	No	[115]
Camembert Blue-veined cheeses	*Candida intermedia*	No	[135]

According to the source of contamination of these potentially pathogenic yeasts, and considering the population segment to be protected, the first preventive measure within the HACCP plan should be the highest level of hygiene in the industries. This fact should be maximized in those kinds of cheeses that have not undergone thermal treatment, or in those industrial stages after this treatment.

### 3.2. Yeast Biocontrol Strategies Using Microorganisms

One of the most commonly used strategies is to exploit the capacity of some yeasts with QPS status, able to grow in ripened foods and endowed with antagonistic activity against other yeasts. Among these yeasts, *D. hansenii* is used in the production of ripened food of animal origin. This antagonistic capacity may be caused by the production of toxic proteins or glycoproteins called killer toxins or mycocins, which can kill sensitive yeast, but can be innocuous for consumers. Mycocin activity has been reported in more than 90 yeast species, and their presence is directly related with the presence of chromosomal or extrachromosomal genes (linear plasmids or viruses) [136]. This behaviour is not uniform among these species, nor can it be linked to sources of isolation [137]. Additionally, this killer phenotype is markedly affected by the substrate physicochemical characteristic where the yeast grows [117].

Several studies have reported the ability of mycocins from foodborne yeasts to kill pathogenic yeasts in vitro [138]. Killer activity by some yeasts against *C. albicans* was reported many years ago. Mycocins from *D. hansenii* have shown activity against opportunistic pathogenic including *Candida* species. [137]. The killer activity of *D. hansenii* against *C. albicans* and *C. tropicalis* in ripened cheeses was demonstrated. Therefore, these observations raise the possibility that *D. hansenii* could hamper *Candida* survival [117]. On the other hand, K. marxianus and Kluyveromyces lactis inhibited the growth of *C. albicans* isolated from “Tomme d’orchies” cheese [139].

LAB have long been used in dairy and meat products, providing microbial safety and organoleptic benefits. There are patented microorganisms, such as *Lacticaseibacillus rhamnosus*, for use as “yeast and mold control”, as well as *L. plantarum*. Some other LAB species, for e.g., *Lactobacillus acidophilus* and *Limosilactobacillus reuteri*, also show the capacity to inhibit yeasts producing metabolites with antifungal activity [116]. In a study conducted by Makki et al. [140], a protective culture combining a mixture of *Lacticaseibacillus* spp. and *Lactiplantibacillus* spp. had an effect on the outgrowth of *D. hansenii*, *Meyerozyma guilliermondii* and *Torulaspora delbrueckii* in “cottage” cheese.

### 3.3. Plant-Derivative Biocontrol Strategies

Concerning the use of plant-derived biocontrol agents, a leaf extract of *Lawsonia inermis*, an Indian herb, showed a very effective anti-candidal activity with differents sites of action, such as germ tube inhibition, protease, phospholipases and aspartate dehydrogenase inhibitory activity [141]. *Solanum lycopersicum* shows high levels of fungistatic activity against *Candida* spp., with its suggested mode of action being the targeting of the *C. albicans* ergosterol pathway via the upregulation of ergosterol genes [142]. A four percent *Jugulans nigra* extract is effective in eradicating *C. albicans* as clotrimazole due to juglone, an active component found in the black walnut tree [143]. The highest antimicrobial activity of clove (*Syzygium aromaticum*) against *C. albicans* was achieved at the concentration of 0.2% by causing damage to fungal membranes and cell walls [144,145]. Papaya seed extracts also cause apoptosis in *Candida* cells due to the oxidative stress created [146], with a similar effect to garlic oil (*Allium sativum*) against *C. albicans* [147]. Aloe vera, oregano leaf and grapefruit seed extract have all been shown to inhibit the growth of *Candida* species as well [116]. The inhibitory effect of *Eugenia caryophyllata* thumb leaf EOs on contaminating microorganisms of “Coalho” cheese was investigated, with the lowest minimum inhibitory concentration (MIC) level (200 µL mL^−1^) against *C. albicans*, *C. parapsilosis* and *C. krusei* being obtained [148]. 

Although these results are promising, it would be necessary to validate these effects of BCAs in food matrices. This should be performed in order to take into account the possible interaction of these agents with each of the ingredients of the cured products, which may modify their antimicrobial capacity. With regard to the effect on the organoleptic characteristics of the product, none of the above studies evaluated the effect on the sensory characteristics of the final product. If these extracts were to be used on an industrial scale, it would be necessary to assess these effects, as these are extracts and plants with a high organoleptic impact.

## 4. Pathogenic and Toxigenic Molds in Ripened Foods and Biocontrol Strategies

Some molds can cause a wide variety of human diseases such as allergic or invasive infections due to excessive inhalation of spores (mainly from *Aspergillus* spp.) or their transmission through infected wounds, as well as through the smoking of contaminated plants [149]. However, these infections are infrequent, and from the food safety view, the main problem associated with the mold contamination of ripened animal products is the production of mycotoxins, which are secondary metabolites with a wide range of toxic effects. The most important mycotoxins in dry-cured meat products are the OTA and AFs, due to their frequency and their toxicity, although other mycotoxins can be detected in these products, such as CPA, STG and CIT [24,25]. Similarly, the abovementioned mycotoxins have also been described in cheeses as well as PR toxin, roquefortine C and patulin [23,150]. The main molds that produce mycotoxins in animal-origin ripening foods are described below.

### 4.1. Biocontrol Strategies against Ochratoxin A-Producing Molds

OTA can be produced by different species of *Penicillium* and *Aspergillus*, such as *Penicillium nordicum*, *Penicillium verrucosum*, *Aspergillus westerdijkiae* and *Aspergillus carbonarius*. Within these species, *P. nordicum* has been described as the main OTA producer in dry-cured meat products and cheeses [151]. This mycotoxin is nephrotoxic, hepatotoxic, teratogenic, immunotoxic and has been classified as a possible human carcinogen (group 2B) by the International Agency for Research on Cancer (IARC) [152,153,154]. Preserved meats and cheeses are the main contributors to dietary exposure to OTA in several European countries [154].

The biocontrol of ochratoxigenic molds employed in dry-cured meat products includes the use of starter and protective cultures which contain LAB, GCC+, yeasts and non-toxigenic molds, as displayed in Table 3 [31,37,155,156,157,158,159,160,161]. Different strains from *Enterococcus faecium* were demonstrated to control OTA production via growing *P. nordicum* in a dry-cured fermented sausage based medium, although they did not affect the OTA produced by *P. verrucosum* [157]. GCC+, as *S. xylosus*, successfully decreased the OTA content using different strains of *P. nordicum* (Pn15, Pn92 and Pn856) a in dry-cured ham-based medium, although no effect was detected in sausages inoculated with the same strain of *S. xylosus* and Pn15 [31,160]. Fermented extracts developed from the fermentation of a meat model system (BFS) by *L. plantarum* and *P. pentosaceus* were able to totally eliminate the presence of *P. nordicum* and *P. verrucosum* using different concentrations depending on the bacterium and the mold strain tested [162]. Meftah et al. [159] revealed the ability of the yeasts *C. zeylanoides* and *Rhodotorula mucilaginosa* to reduce the OTA concentration produced by *P. nordicum* and *A. westerdijkiae* in three matrices (ham, and dry-cured sausages with industrial and traditional processing). Other yeasts, such as *D. hansenii* and *Saccharomycopsis fibuligera*, were able to completely inhibit the OTA produced by *P. nordicum* and *A. ochraceus* in speck, a typical meat product in the European Alpine area [37]. *D. hansenii* has also been displayed as an effective BCA against *P. nordicum*, *P. verrucosum* and *A. westerdijkiae* tested in other studies in dry-cured meat products or meat-model systems [31,159,161,163,164]. Additionally, the strain of *D. hansenii* used in some of these studies did not negatively modify the sensorial quality of dry-cured fermented sausages which contained it [30]. The non-toxigenic mold *Penicillium chrysogenum*, producer of the antifungal protein PgAFP, was proposed as a BCA against *P. nordicum* in a dry-cured ham-based medium [158] and in a meat-model system [165], showing in both studies possible nutrient competition. Similarly, this strain of *P. chrysogenum* controlled the growth of potentially ochratoxigenic molds, reducing the OTA accumulation in dry-cured Iberian hams which had undergone industrial processing [166]. This strain was also proposed as a good protective culture with no technological drawbacks during the ripening of dry-cured fermented sausages [30]. The protective potential of a commercial starter culture of *Penicillium nalgiovense* was displayed by the decrease in the OTA concentration produced by *P. verrucosum* in the dry-cured fermented sausage “salchichón” [167].

Regarding the use of plant derivatives as biocontrol agents, some studies demonstrated the efficiency of reducing OTA using ingredients from dry-cured meat sausages such as rosemary, oregano and smoked paprika “pimentón” [41,44]. Oregano and rosemary leaves added to a dry-cured fermented-sausage medium and “pimentón” to a meat-based medium decreased the amount of OTA produced by *P. nordicum* [41,44]. Rosemary leaves were able to decrease the mycotoxin produced by *P. nordicum* during dry-cured sausage ripening and, together with their essential oil, the OTA produced by *A. westerdijkiae* in a dry-cured fermented sausage based medium [40,163]. However, the sensorial impact of these BCAs was not checked, although the concentrations of herb leaves used were expected to have no negative influence on consumer perceptions [41,163]. Additionally, other EOs such as basil EO, sage EO and oregano EO, and plant derivatives such as carvacrol and eugenol, have also been described as BCAs against ochratoxigenic molds in commercial culture media, but not in meat-based matrices [175,176], therefore its effectiveness in ripening products is not known yet.

On the other hand, there are few studies focused on the biocontrol of ochratoxigenic molds in cheeses, despite the fact that all kinds of ripened cheeses can be contaminated with this mycotoxin [150,168,177,178]. The use of LAB such as *Lactobacillus buchneri* and *L. casei* against *P. nordicum* in cheeses covered with films with the bacteria incorporated reached OTA reductions of up to 94%, although no sensory study was carried out to confirm their applicability [168]. Another study, which employed twenty-five strains of *L. plantarum*, one *Lacticaseibacillus paracasei*, one *L. casei* and one *L. rhamnosus* isolated from a Brazilian artisanal “Serrano Catarinense” cheese, showed the ability of these LAB to decrease the growth of *P. nordicum* in MRS agar, suggesting a possible future use as preservative agents during cheese manufacturing [179]. The lack of studies about the biocontrol of ochratoxigenic molds in cheeses opens up a new field of study necessary to reduce the risk posed by OTA presence in these ripened products.

### 4.2. Biocontrol Strategies against Aflatoxin-Producing Molds

AFs are highly toxic secondary metabolites produced by molds such as *Aspergillus flavus*, *Aspergillus parasiticus* and *Aspergillus nomius* [180]. Although these fungi are more frequent in cereal crops, they can colonize the surface of ripening products of animal origin [181,182,183]. The most important AFs are B1, B2, G1 and G2. These mycotoxins are carcinogenic (Group 1) and mutagenic for animals and humans according to the IARC [152].

Similar to the strategies employed against ochratoxigenic molds, different LAB, GCC+ and yeasts have been studied as BCAs against AF producers in dry-cured meat products. *S. xylosus* Sx8 was able to control the growth and the AFB1 produced by *A. flavus* and AFB1 and AF G1 produced by *A. parasiticus* in a dry-cured ham-based medium at three different temperatures (15, 20 and 25 °C) [160]. The BFS extract from *L. plantarum* and *P. pentosaceus* reduced the growth of *A. flavus* and *A. parasiticus* in a meat model system by up to 50% using concentrations between 21 and 43 g L^−1^ [162]. *D. hansenii* combined with the antifungal protein PgAFP and *P. acidilactici* on slices of dry-cured fermented sausages successfully diminished AFB1 and AFG1 amounts produced by *A. parasiticus* and the mold counts [169]. In another study, *D. hansenii* was tested against *A. parasiticus* and decreased the AF B1 in more than 53.85% and the AFG1 by up to 59.06% in dry-fermented sausages, while the AFG1 was below the limit of quantification in dry-cured ham [39].

Regarding the agents of plant origin, the smoked paprika “pimentón” reduced the AFB1 and AFG1 production by *A. parasiticus* in a dry-cured meat model system, although they did not decrease the mold’s growth [44].

The presence of AFs in cheeses has been described worldwide. In addition to the AFM1 that can be present in the milk used for cheese manufacturing, common aflatoxins have been detected due to the surface colonization of the product by aflatoxigenic molds [23,184,185,186]. Despite the risk that AFs pose in cheeses, there are only a few studies based on the biocontrol of AFs in this matrix. In cheese slices, the use of the protein PgAFP combined with *D. hansenii* in the presence or absence of *P. acidilactici* decreased the *A. parasiticus* growth and its AFG1 production below the method’s limit of detection [169]. On the other hand, the addition of *Oreganum vulgare* EO (0.02% *v*/*v*) to the Minas cheese formulation inhibited the germination of spores of *A. flavus* for up to 15 days of ripening, and was the cheese flavor and taste accepted by the panelists [170]. Moreover, Vitalini et al. [171] demonstrated that parsley EO applied on cheese slices was effective in preventing *A. flavus* growth. Tatlisu et al. [45] demonstrated the antifungal activity of thymol (main component of numerous EOs) and nanofibers with thymol applied to “kashar” cheese cube surfaces against *A. parasiticus*, although no sensory analyses was performed [45].

### 4.3. Biocontrol Strategies against Cyclopiazonic Acid-Producing Molds

CPA can be produced by different molds, such as *A. flavus*, *A. parasiticus* and *Penicillium griseofulvum*, in ripened meats, and mainly *P. commune*, *P. roqueforti* and *P. camemberti* in cheeses [24,182,187]. Due to the little amount of toxicological data, the IARC has not declared an acceptable CPA toxicity level yet, but it is well known that it includes sever gastrointestinal and neurological disorders and organ necrosis [188]. Therefore, this lack of data and legal limits results in a shortage of studies about the biocontrol of molds that only produce CPA.

Concerning the biocontrol studies in dry-cured meat products, the bacterium *S. xylosus* Sx8 decreased CPA production using two strains of *P. griseofulvum* grown in a dry-cured ham-based medium after 30 days of incubation at 25 °C [160]. Concentrations of 85 g L^−1^ of BFS extract from *L. plantarum* and between 21 and 85 g L^−1^ from *P. pentosaceus* did not allow for the growth of *P. griseofulvum* and *P. commune* in a meat model system [162]. Moreover, Delgado et al. [34] showed that *P. chrysogenum*, producer of the antifungal protein PgAFP, was able to diminish CPA amounts produced by *P. griseofulvum* under the limit of detection on a dry-fermented sausage-based medium and more than 97% on dry-cured fermented sausages after 21 days following industrial ripening [34].

In Edam cheeses, the clove, thyme, red thyme and litsea EOs completely inhibited the growth of two CPA producer strains of *P. commune*, while cumin and marjoram EOs showed high antifungal activity, although they did not totally inhibit the growth of the molds [43]. In this study, the evaluators recognized some EOs in sensory evaluation via the triangle test, but they did not have a negative effect on the taste and smell of the treated cheeses [43].

### 4.4. Biocontrol Strategies against Sterigmatocystin-Producing Molds

STG is a precursor of AFB1, so the producing molds mainly include different *Aspergillus* species, although there are other ones such as *Eurotium*, *Fusarium* and *Podospora* spp., which demonstrate an ability to produce this mycotoxin [189]. Concerning its toxicity, it has been found that STG induces tumors in animals and humans [189]. In spite of this evidence, the IARC only classified STG into Group 2B (possible human carcinogen) [190].

Regarding the biocontrol strategies, the BCA against AF producers could be applied for the toxigenic molds which produce both mycotoxins (AFs and STG), but STG production deserves to be further studied. Within the ripened animal products, STG has been mainly described in a wide range of cheeses contaminated with *Aspergillus versicolor*, *A. flavus* or *A. parasiticus* [189,191]. However, there are no studies of ripened matrices of animal origin based on the biocontrol of molds that only produce STG, although EOs from tarragon, oregano and savory showed the inhibition of two producers of STG isolated from cheeses, *Aspergillus puulaauensis* and *Aspergillus jenseii* [192].

### 4.5. Biocontrol Strategies against Citrinin-Producing Molds

CIT is produced by different species of *Penicillium* and *Aspergillus*, including *P. cambemberti*, *Penicillium expansum*, *P. verrucosum*, *P. citrinum*, *P. viridicatum Aspergillus carneus* and *Aspergillus niveus* [23]. Several studies have shown frequent cooccurrence of OTA and CIT in dry-cured meat products and cheeses [25,193]. CIT is nephrotoxic and hepatotoxic to humans and has been classified into Group 3 by the IARC [190] due to evidence of its in vivo carcinogenicity [194,195].

Given that some toxigenic strains can produce both OTA and CIT, the strategies to prevent OTA producers may also be effective for CIT accumulation. However, it must be considered that some molds can switch the production of OTA to CIT or vice versa to deal with different stressful environments [196,197]. Therefore, different strategies might be needed for reducing both mycotoxins. To our knowledge, there are no studies only focused on the biocontrol of this mycotoxin in dry-cured meat products. However, in cheeses a concentration of 150 µg mL^−1^ of eugenol and thymol inhibited CIT production by *P. citrinum* in “Arzúa-Ulloa” cheese, while in “Cabreriro” cheese these antifungal agents did not affect the CIT amounts [172]. In other studies, the *Zataria multiflora* Boiss EO decreased the growth and CIT production by *P. citrinum* in Iranian cheese and mozzarella [173,174]. Despite the use of EOs and compounds with a strong flavor in the above-mentioned studies, only the organoleptic effect of *Z. multiflora* Boiss EO was tested. Concentrations over 600 ppm, which were more effective against CIT production, were disliked by the consumers and, consequently, their applications were limited [174].

Despite the studies about the presence of mycotoxins in ripened products of animal origin, no notifications were made regarding the presence of mycotoxins in both meat and dairy products (RASFF).

## 5. Challenges in the Application of Microorganisms as Biocontrol Agents in Animal Origin Ripened Foods

The successful utilization of microorganisms as BCAs requires their effective growth and development in/on the food matrix, where it will be confronted with the targeted pathogenic microorganisms.

The isolation of autochthonous microorganisms from these products to be used as protective cultures is then of utmost interest since these strains are well adapted to these specific ecological niches. This ensures their survival and competition under processing and/or storage conditions. If these microorganisms are not isolated from the product intended to be inoculated, they should be examined to check their survival and development under the specific ripening conditions to select or discard them.

In order to maximize the correct implantation of microorganisms previously isolated from ripened products to be used as biocontrol strategies, prior *in vitro* tests are required. These *in vitro* tests are carried out by simulating the environmental conditions given in the different stages in which these microorganisms are inoculated during the ripening process [71]. Within these conditions we find intrinsic factors of the product, such as a_w_ [164], pH [198], salt concentration [199] and interaction with other ingredients [165,200]; and conditions given in the ripening chambers, such as temperature [157] and relative humidity. All these assays should be performed with the aim of identifying the most appropriate stage, throughout the ripening process, for their inoculation as BCAs.

**Figure 1:**
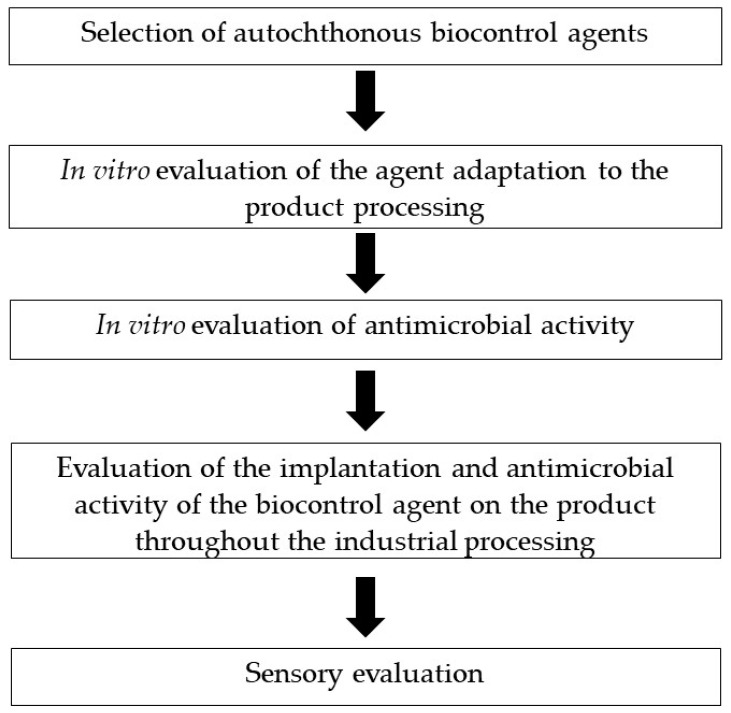
Flow chart of the biocontrol agents’ selection and evaluation, a process to be used for ripened foods of animal origin.

After this, the next step aims to evaluate its biocontrol potential via co-inoculation with the target pathogen microorganism in food models simulating temperature, NaCl concentration, a_w_ and pH conditions of ripened foods, as displayed in Figure 1. This can be achieved by using culture media which simulates dry-cured ham, dry-fermented sausage or ripened cheese [39,41,85,160].

Furthermore, the use of a model system to study the mechanisms and modes of action of BCAs is a powerful tool to avoid the effect of external factors, which could hide or modify the metabolic routes involved in their antimicrobial effect [201,202]

Once the inhibitory capacity of a BCA in a culture medium has been proven, it is necessary to evaluate it in the ripened foods of animal origin following their industrial processing. For this purpose, different studies have, firstly, inoculated the BCAs at the beginning of the product manufacturing and tested their antimicrobial activity [34,38,68,163] and, secondly, tested the BCA implantation at the end of the ripening [31]. Different molecular techniques are available to check whether the protective microorganisms have been correctly implanted in the product, such as analysis of chromosomal DNA via pulsed field gel electrophoresis (PFGE). This methodology has been reported as appropriate for the differentiation of LAB, GCC+ and yeast strains [31,203]. In the same way, RAPD-PCR analysis or the use of mitochondrial DNA restriction patterns have also been used to discriminate yeasts at the strain level [204,205]. In addition, RAPD-PCR analysis has also been proven to be suitable for the identification of mold strains [206].

Finally, to evaluate the suitability of protective microorganisms as preventative measure for HACCP, the performance of challenge tests during industrial ripening could give additional information in order to assess the actual efficiency of any BCA against the targeted pathogenic/toxigenic microorganism under the real ripening conditions in foods of animal origin [34,77].

## 6. Conclusions

The current microbial hazards found in ripened foods of animal origin require constant control from the food industry, since food safety is a primary requirement for the global food market. Not only are safe products demanded by consumers, but the avoiding of the use of chemical preservatives to manage these pathogenic and toxin-producer microorganisms is also wanted. The successful effect of microorganisms as BCAs requires their correct development on the foodstuffs throughout the industrial ripening process. Different strategies to counteract worrying foodborne pathogens have been evaluated, displaying apparent positive results. Their ability to inhibit pathogenic microorganisms or their toxins has been tested to different extents. Finally, their minimal or absent sensory impact must be also fulfilled prior to being included as preventive measures in the HACCP programs.

## Figures and Tables

**Table 1:** Reduction of pathogenic bacteria viability using biocontrol agents in ripening food matrices of animal origin.

Matrix	Pathogenic Bacteria	Biocontrol Agent	Pathogenic Bacteria Viability Reduction	Reference
Spanish dry-cured fermented sausages	*Listeria monocytogenes*	*Latilactobacillus sakei* 197	1.77 log CFU g^−1^	[65]
Short-ripening Spanish traditional cheeses	*L. monocytogenes*	*Lacticaseibacillus casei* 116	2.2 log CFU g^−1^	[66]
Brazilian artisanal cheeses	*L. monocytogenes* and *Staphylococcus aureus*	*Lactiplantibacillus plantarum* (1QB77)	3 and 2.3 log CFU g^−1^, respectively	[67]
Frescal and semi-hard artisanal Minas microcheeses	*L. monocytogenes*	*Levilactobacillus brevis* *L. plantarum* *Enterococcus faecalis*	Inactivation	[36]
Dry-fermented Greek sausage	*L. monocytogenes* and *Escherichia coli*	*L. sakei* 8416 *L. sakei* 4413	2.2 log CFU g^−1^	[68]
Bryndza cheese	*Salmonella enterica* and *S. aureus*	*Lactococcus lactis* *Lactiplantibacillus paraplantarum*	Significative reduction	[69]
Traditional dry-fermented sausage suçuk	*S. aureus*	*L. sakei* *Staphylococcus carnosus*	More than 2 log CFU g^−1^	[70]
		*Pediococcus acidilactici*, *Latilactobacillus curvatus*, *Staphylococcus xylosus*	More than 2 log CFU g^−1^	
Fermented salami	*Clostridium perfringens* and *Clostridium* species	*L*. *plantarum* PCS20, *Lactobacillus delbrueckii* DSM 20074	2 and 1.5 log CFU g^−1^, respectively	[71]
Dry fermented sausages	*E. coli*, *L. monocytogenes*, *Salmonella* spp. and sulfite-reducing clostridia	*Juniperus communis* L. Essential oil (EOs)	Inactivation	[72]
Dry cured sausage ‘Chouriço de vinho’	*Salmonella* spp., *L. monocytogenes* and *S. aureus*	Bay, garlic, nutmeg, oregano, rosemary and thyme EOs	0.1–2 log CFU g^−1^	[42]
Dry cured sausage ‘Chouriço de vinho’	*S. aureus*	Thyme EOs	Inactivation	[42]
Cured ham-based medium	*L. monocytogenes*	Cinnamon, pomegranate and strawberry extracts	3 log CFU mL^−1^	[73]

**Table 3:** Main effects of biocontrol agents on toxigenic molds in ripening food matrices of animal origin.

Ochratoxin A (OTA) Producers				
Matrix	Mold	Biocontrol Agent	Effect on Growth or Mycotoxin Production	Reference
Dry-cured fermented sausage medium	*Penicillium nordicum*	*Enterococcus faecium*	↓ ^1^ OTA ↓ Growth	[157]
Dry-cured ham based medium	*P. nordicum*	*Staphylococcus xylosus*	↓ OTA ↓ Growth	[160]
Fermented meat model system	*P. nordicum* and *Penicillium verrucosum*	*Lactiplantibacillus plantarum* and *Pediococcus pentosaceus*	↓ Growth	[162]
Ham, industrial and traditional dry-cured sausages	*P. nordicum* and *Aspergillus westerdijkiaie*	*Candida zeylanoides* and *Rothia mucilaginosa*	↓ OTA ↓ Growth	[159]
Speck	*P. nordicum* and *Aspergillus ochraceus*	*Debaryomyces hansenii* and *Saccharomycopsis fibuligera*	↓ OTA ↓ Growth	[37]
Dry-cured fermented sausages	*P. nordicum*	*D. hansenii*	↓ OTA	[31,161,163]
Dry-cured meat model systems	*P. verrucosum*, *P. nordicum*	*D. hansenii*	↓ OTA ↓ Growth	[156,164]
Dry-cured fermented sausage medium	*A. westerdijkiae*	*D. hansenii*	↓ OTA	[155]
Dry-cured ham based medium	*P. nordicum*	*Penicillium chrysogenum*	↓ OTA	[158]
Meat model system	*P. nordicum*	*P. chrysogenum*	↓ OTA ↓ Growth	[165]
Dry-cured Iberian ham	Wild mycobiota containing OTA producer strains	*P. chrysogenum*	↓ OTA	[166]
Dry-cured fermented sausage	*P. verrucosum*	*P. nalgiovense*	↓ OTA	[167]
Dry-cured fermented sausage medium	*P. nordicum*	Rosemary and oregano leaves	↓ OTA	[30]
Dry-cured fermented sausage medium	*P. nordicum*	Smoked paprika “pimentón”	↓ OTA	[44]
Dry-cured fermented sausages	*P. nordicum*	Rosemary leaves	↓ OTA	[163]
Dry-cured fermented sausage medium	*P. nordicum*	Rosemary leaves and rosemary essential oil (EO)	↓ OTA	[40]
Cheeses	*P. nordicum*	*Lactobacillus buchneri* and *Lacticaseibacillus casei*	↓ OTA	[168]
**Aflatoxins (AFs) producers**				
Dry-cured ham based medium	*Aspergillus flavus*	*S. xylosus*	↓ AFB1 ↓ Growth	[160]
Dry-cured ham based medium	*Aspergillus parasiticus*	*S. xylosus*	↓ AFB1 and AFG1 ↓ Growth	[160]
Fermented meat model system	*A. flavus* and *A. parasiticus*	*L. plantarum* and *P. pentosaceus*	↓ Growth	[162]
Dry-cured fermented sausages slices	*A. parasiticus*	*D. hansenii* + PgAFP + *Pediococcus acidilactici*	↓ AFB1 and AFG1 ↓ Growth	[169]
Dry-cured fermented sausages; dry-cured ham	*A. parasiticus*	*D. hansenii*	↓ AFB1 and AFG1	[39]
Dry-cured meat model system	*A. parasiticus*	Smoked paprika“pimentón”	↓ AFB1 and AFG1	[44]
Cheese slices	*A. parasiticus*	*D. hansenii* + PgAFP + *P. acidilactici*	↓ AFG1 ↓ Growth	[169]
Minas cheese formulation	*A. flavus*	*Origanum vulgare* EO	↓ Spores germination	[170]
Cheese slices	*A. flavus*	Parsley EO	↓ Growth	[171]
Kashar cheese	*A. parasiticus*	Thymol	↓ Growth	[45]
**Cyclopiazonic acid A (CPA) producers**				
Dry-cured ham-based model	*Penicillium griseofulvum*	*S. xylosus*	↓ CPA	[160]
Fermented meat model system	*P. griseofulvum* and *Penicillium commune*	*Lc. plantarum* and *P. pentosaceus*	↓ Growth	[162]
Dry-fermented sausage-based medium; dry-fermented sausages	*P. griseofulvum*	PgAFP	↓ CPA	[34]
Edam cheese	*P. commune*	Clove, thyme, red thyme, litsea, cumin and marjoram eOs	↓ Growth	[43]
**Citrinin (CIT) producer**				
Arzúa-Ulloa cheese	*Penicillium citrinum*	Eugenol and thymol	↓ CIT	[172]
Iranian and mozzarella cheeses	*P. citrinum*	*Zataria multiflora* Boiss EO	↓ CIT	[173,174]

^1^ Arrows indicate reduction in growth, mycotoxin production or spore germination.

## Data Availability

Data sharing not applicable.
